# Characterization, Polymorphism and Selection of Major Histocompatibility Complex (MHC) DAB Genes in Vulnerable Chinese Egret (*Egretta eulophotes*)

**DOI:** 10.1371/journal.pone.0074185

**Published:** 2013-09-03

**Authors:** Zeng Wang, Xiaoping Zhou, Qingxian Lin, Wenzhen Fang, Xiaolin Chen

**Affiliations:** 1 Key Laboratory of Ministry of Education for Coast and Wetland Ecosystems, School of Life Sciences, Xiamen University, Xiamen, Fujian, People’s Republic of China; 2 Key Laboratory of Ministry of Education for Coast and Wetland Ecosystems, College of the Environment and Ecology, Xiamen University, Xiamen, Fujian, People’s Republic of China; Kyushu Institute of Technology, Japan

## Abstract

The major histocompatibility complex (MHC) is an excellent molecular marker for the studies of evolutionary ecology and conservation genetics because it is a family of highly polymorphic genes that play a key role in vertebrate immune response. In this study, the functional genes of MHC Class II B (DAB) were isolated for the first time in a vulnerable species, the Chinese egret ( *Egretta* *eulophotes* ). Using a full length DNA and cDNA produced by PCR and RACE methods, four potential MHC DAB loci were characterized in the genome of this egret and all four were expressed in liver and blood. At least four copies of the MHC gene complex were similar to two copies of the minimal essential MHC complex of chicken, but are less complex than the multiple copies expressed in passerine species. In MHC polymorphism, 19 alleles of exon 2 were isolated from 48 individuals using PCR. No stop codons or frameshift mutations were found in any of the coding regions. The signatures of positive selection detected in potential peptide-binding regions by Bayesian analysis, suggesting that all of these genes were functional. These data will provide the fundamental basis for further studies to elucidate the mechanisms and significance of MHC molecular adaptation in vulnerable Chinese egret and other ardeids.

## Introduction

The major histocompatibility complex (MHC) is a multi-gene family, representing an important component in the acquired immune system of the vertebrate species. Traditionally, MHC genes are classified into two major classes, designated class I and class II. The MHC class II genes consists of two genes, named A and B, in which the B genes are responsible for the majority of the polymorphism. MHC is a favored molecular marker for evolutionary ecology and conservation genetics because of their diverse functions and characteristics relevant to evolutionary and adaptive processes [1–6].

Studies on the functional genes of MHC II B (DAB) complex in birds have indicated that multiple copies of these genes are highly variable among different species [5,7–18]. The most complex of the MHC structures were found in passerine birds [19–21] while the “minimal essential” structures were found in a non-passerine bird, the red jungle fowl (Galliformes, Phasianidae, *Gallus gallus*) [22,23]. Other non-passerine birds, including raptors and seabirds, paleognath birds, and penguins have been reported to have more complex MHC structures than the “minimal essential” genes [24].

Efficient and reliable genotyping of MHC genes is a difficult task, yet remains a basic prerequisite to understand the MHC complex in these species [13,16,25]. Target loci are commonly expressed as multiple copies, and allele sequences, even at a single locus, may be very divergent. This makes for a more challenging problem to identify all alleles carried by an individual and reconstruction of multilocus genotype in an individual. The presence of both expressed loci and pseudogenes further complicates the problem in identifying functional variants [14,20,26]. Moreover, the copy number of MHC genes in birds is highly variable. Therefore, the precise genotyping and full understanding of the molecular architecture of the multilocus MHC complex remains an important and challenging problem [12,27].

The Chinese egret (Ciconiiformes, Ardeidae, *Egretta* *eulophotes* ) is listed as a vulnerable species with an estimated global population of 2600–3400 individuals [28,29]. This egret is a migratory colonial waterbird, wintering in the south of Asia and breeding on offshore islands in Russia, North Korea, South Korea and China. This breeding pattern may facilitate pathogen transmission and advance MHC polymorphisms [24]. Several molecular studies have been carried out on this species [30–32], including reports of MHC and two DNA sequences of the DAB gene from exon 2 to exon 3 but these studies did not confirm their expression [33]. The aims of the present study were to: (1) isolate the complete MHC DAB genes in the Chinese egret and determine their architecture; (2) confirm the expression of all the loci; (3) estimate variability in each locus and test to determine if positive selection was acting on the MHC DAB genes; and (4) to investigate the usefulness of noncoding sequence variation for the design of locus-specific amplification strategies. Successful completion of these objectives will provide the essential fundamentals for further studies to determine the mechanism and significance of MHC molecular adaptation in vulnerable Chinese egret and other ardeids.

## Materials and Methods

### Ethics Statement

This research and all procedures involving collection of animal tissue in the wild were approved by the Administration Center for Wildlife Conservation in Fujian Province (FJWCA-1208). The scientific license for access to the study site was issued by the Administration Department of Xiamen Egret Natural Reserve (XMENR-1005). Blood samples (~0.5 mL) were collected from 32 nestlings (aged around 15 days) of *E* *. eulophotes* by puncturing the wing vein and using a syringe to draw up the blood. The nestlings were immediately returned to the nest after stanching the blood with cotton. Collection of the blood sample was conducted during the morning, and visits to the breeding colony were restricted to a maximum of two hours per day.

### Methods for DNA Extraction, RNA Extraction and Reverse Transcription

Blood samples were collected from 48 nestlings (aged around 15 days) of the Chinese egret on the Riyu Islet (27°01′N, 120°25′E) in Fujian Province of China as described above, and frozen at −80°C until DNA extraction. Total genomic DNA was isolated using the Universal Genomic DNA Extraction Kit Ver. 3.0 (TaKaRa), and total RNA was extracted from the blood and from the liver of one individual Chinese egret that died at this Islet by Trizol (Invitrogen, Switzerland), respectively. 

### Isolation of MHC DAB Genes

A fragment from exon 1 to exon 3 of the MHC DAB gene complex in the Chinese egret was first amplified with degenerate primers 34F and Rap3R [11]. PCR reactions were carried out on a Biometra T gradient thermocycler in a final volume of 20 µL containing 1 × PCR buffer (50 mM KCl, 10 mM Tris-HCl, pH 8.3, 1.5 mM MgCl_2_), 0.2 mM of each dNTP, 0.4 µM of each primer, 0.7 U of Taq polymerase (TaKaRa) and 100 ng of genomic DNA. The conditions for PCR amplification were a denaturing step at 94 °C for 3 min, followed by 35 cycles at 94 °C for 30 s, 55 °C for 30 s, 72 °C for 2 min, and a final extension at 72 °C for 10 min.

The 3’ and 5’ rapid amplification strategy of cDNA ends (RACE) [34] was used to isolate the entire gene sequence and confirm the gene transcription by using 3’-full RACE core set ver. 2.0 (TaKaRa) and 5’-full RACE Kit (TaKaRa). Based on the DNA sequence obtained from the fragment, gene specific outer/inner primers were designed in conserved exon 3 regions for 3’ RACE (23sp1: ACAGGCTGGTTTGCTACGTGACG 23sp2: CGGCGGAGAT TGAGGTGAAGTGG) and 5’ RACE (25sp1: CCACCTGGCACATGTAG GTGTCC 25sp2: TGGTTTCCAGCATCACCAGCACCTG) respectively, using Oligo 6.0 (Molecular Biology Insights). PCR reactions were carried out following the protocols and applications as described in the kit. A 580 bp segment and a 650 bp fragment were obtained from the 3’ RACE and 5’ RACE, respectively. After removing the overlapping region, a 1060 bp long cDNA segment of the MHC DAB genes from the 5’ UTR to the 3’ UTR was obtained.

According to the complete cDNA sequence, two primers 25utrF：CAGAACTCTGCCCGGAGACGG and 23utrR：CTGCAGAGCAGCGACAG CGAA were designed to amplify the complete DNA sequence of the MHC DAB gene. PCR reactions were carried out on a Biometra T gradient thermocycler in a final volume of 50 µL containing 1× LA PCR buffer II, 2.5 mM MgCl_2_, 0.4 mM dNTP, 0.5 µM of each primer, and 2 U TaKaRa, LA Taq. PCR conditions included an initial denaturation step at 95°C for 3 min, 35 cycles of denaturation at 95°C for 30 s, annealing at 62°C for 30 s, primer extension at 72°C for 3 min, and a final extension at 72 °C for 10 min.

To verify differences among the four MHC DAB loci in the intron 1 and locate the conserved regions to design locus-specific primers, two primers 25utrF1: AGCTCATTTCGGGAGGGGGTC and 23exr: CATCACCAGCAC CTGGTAGGTCC were used to amplify a fragment from the 5’ UTR to exon 3 for the 32 nestlings randomly selected. PCR reactions were carried out in a final volume of 20 µL containing 1 × PCR buffer (50 mM KCl, 10 mM Tris-HCl, pH 8.3, 1.5 mM MgCl_2_), 0.2 mM of each dNTP, 0.4 µM of each primer, 0.7 U of Taq polymerase (TaKaRa) and 100 ng of genomic DNA. The condition for PCR amplification included a denaturing step at 94 °C for 3 min, followed by 35 cycles of 94 °C for 30 s, 62 °C for 30 s, 72 °C for 2 min, and a final extension at 72 °C for 10 min. All PCR products were purified using the Agarose Gel DNA Purification Kit Ver. 2.0 (TaKaRa), then ligated into pMD 18-T vector (TaKaRa) and transformed into *Escherichia coli* DH5α. Ten positive colonies of each band were selected to sequence bidirectionally on an automatic sequencer (ABI PRISM 3730; Invitrogen Biotechnology Co. Ltd.) using universal M13 sequencing primers and BigDye version 3 (Applied Biosystems).

### Polymorphism of the MHC DAB Gene Complex

To detect polymorphism within the MHC DAB genes in wild populations of the Chinese egret, 48 nestlings on the Riyu Islet were genotyped by semi-nested PCR combined with single strand conformation polymorphism (SSCP). First, four forward locus-specific primers in intron 1 were designed and designated as DAB01F-04F(DAB01F: GAGGTGCTG GGTGTGGGTGG, DAB02F: GAGGTGCTGGGTGTGGGAATGG, DAB03F: CACTACCAGGACA CGGCTTGTGG and DAB04F: CTACAATTCCAGTTTAA GTGCGACTG). These were combined with the reverse primer DAB2exR: CCACGT GCTCACCTCTCCTATCC to amplify the four loci, respectively. PCR was carried out in a 20 µL reaction mixture containing 1 µL genomic DNA, 0.7 U of Taq polymerase (TaKaRa), 1.5 mM MgCl_2_, 200 µM of each dNTP and 0.4 µM of each primer, for 25 cycles at 94°C for 30 s, 58°C for 30 s and 72°C for 1min. Second, to obtain suitable length fragments for SSCP genotyping and scanning of the variation in exon 2 of each locus, a second round PCR as carried out using the primer DAB2exF: CTGCACAAACAGGG GTTTTCCAG. DAB2exR was used to amplify the entire exon 2 in each locus. The reaction conditions for the second round PCR were identical with those described for the first round. The PCR products from first round were diluted 100-fold and used as the template. The amplicons were separated by SSCP analyses as described by Fain [35]. Five µL of each PCR product were mixed with an equal volume of 95% formamide, denatured for 5 min at 99°C and immediately chilled on ice water for 10 min. The reaction mixture was loaded on an 8% nondenaturing polyacrylamide gel (PAGE) (37.5:1). Electrophoresis was carried out at 10°C for 5 h at 8 W per gel followed by a sensitive silver-staining procedure, as described by Budowle [36]. Samples with equal banding patterns were rearranged and electrophoresed a second time on a nondenaturing PAGE on adjacent lanes to ensure the genotyping, and samples with unique genotypes, were typed using two independent PCR reactions. All SSCP-bands were cut from at least two individuals of each genotype and incubated in 80 µL water for at least 3 h at 37°C. One µL was used as template in a second PCR under the same conditions as described above. The products were screened in an additional SSCP analysis and every allele was directly sequenced in both directions from at least two individuals or two independent PCRs from one individual.

### Exclusion of PCR Errors and Definition of Alleles

For all PCRs, the amplification, cloning or sequencing was carried out twice and only sequences found in both experiments were included in the analysis to avoid the inclusion of PCR artifacts. Since recombination of cloned PCR products can introduce additional artifacts [37,38], direct sequencing of uncloned PCR products was used for agreement with the polymorphic sites. The sequence obtained from SSCP was directly sequenced in both directions. Only completely identical sequences found in two independent PCR events were defined as alleles. These alleles were referred to as “verified” because the same sequence could not arise twice from independent amplification errors. Throughout this study, the word “allele” is used to describe a 270 bp exon 2 sequence derived from SSCP genotyping.

### Data Analyses

The complete DNA and cDNA sequences for MHC DAB, derived from the Chinese egret, were aligned using the DNAMAN software package (Lynnon Biosoft, Quebec, Canada). Exons and introns were distinguished using the GenScan Program [39]. DNA fragments obtained from the 32 nestlings were edited in the BioEdit Sequence Alignment Editor [40]. Alignment gaps were treated as missing data in all analyses. Sequence variability statistics were calculated using the Mega 4.0 Program [41]. The proportion of synonymous (d_S_) and non-synonymous (d_N_) substitutions were calculated for both the 24 possible peptide-binding regions (PBRs) as defined by Brown [42], and the remaining non-antigen binding sites (non-PBRs) for each of the four loci. The Z-Test in the Mega 4.0 was used to test for directed selection. Standard errors were obtained with 1,000 bootstrap replicates, including average rates of synonymous (d_S_) and nonsynonymous (d_N_) substitutions per site according to the Nei–Gojobori method with Jukes–Cantor correction for multiple substitutions [43]. Population allele frequencies and tests of deviation from Hardy–Weinberg equilibrium were calculated using GENEPOP 4.0 [44]. The programme CODEML in the PAML package, version 3.15 [45] was used to test for the presence of codon sites affected by positive selection and to identify those sites in exon 2 sequences of the Chinese egret, and the neutral model M7 and the positive selection model M8 were compared using maximum likelihood ratio tests (LRT).

## Results

### Characterization of MHC DAB Gene Complex

**Figure 1:**
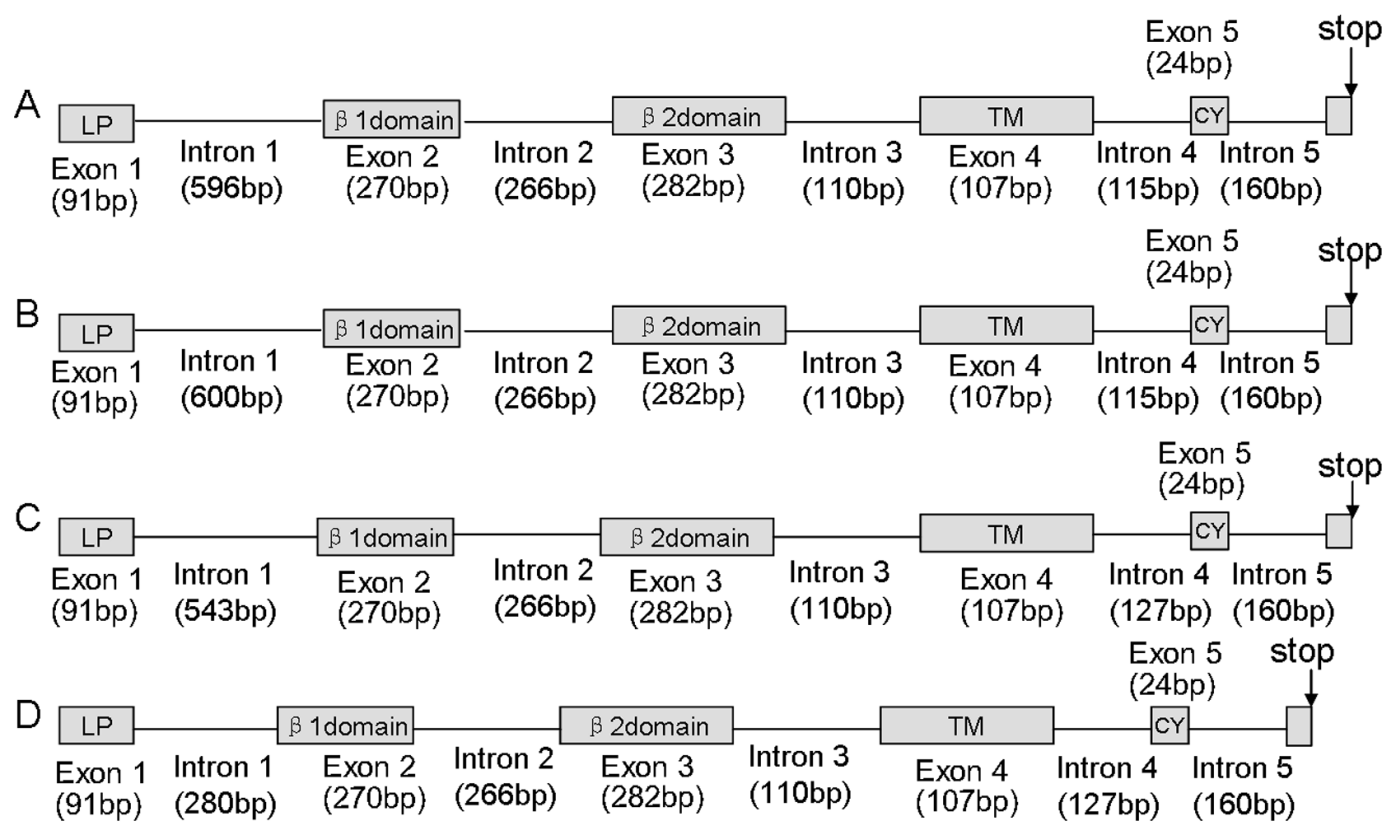
Schematic illustration of MHC II B genes in the Chinese egret. Egeu-DAB1, Egeu-DAB2, Egeu-DAB3 and Egeu-DAB4 are labeled as A, B, C and D respectively. Exons are represented in boxes. Functional domains are indicated in light grey. The highly divergent regions encompassing intron 1. LP, leader peptide; TM, trans-membrane domain; CY, cytoplasmic domain.

In this study, DNA sequences of four different MHC DAB genes were isolated from one individual Chinese egret, which was highly similar over large stretches but distinguished by the divergent intron 1. According to Klein [46], these four genes were designated as Egeu-DAB1, Egeu-DAB2, Egeu-DAB3 and Egeu-DAB4 (GenBank Accession KC282841-KC282844). The result of RACE analyses indicated that each of the four genes comprised a total of 780 bp encoding for 260 amino acid residues sequences in exons. This encoded for six of the expected exons, including the signal peptide, the β1, β2, transmembrane proteins and the cytoplasmic domains (Figure 1).

**Figure 2:**
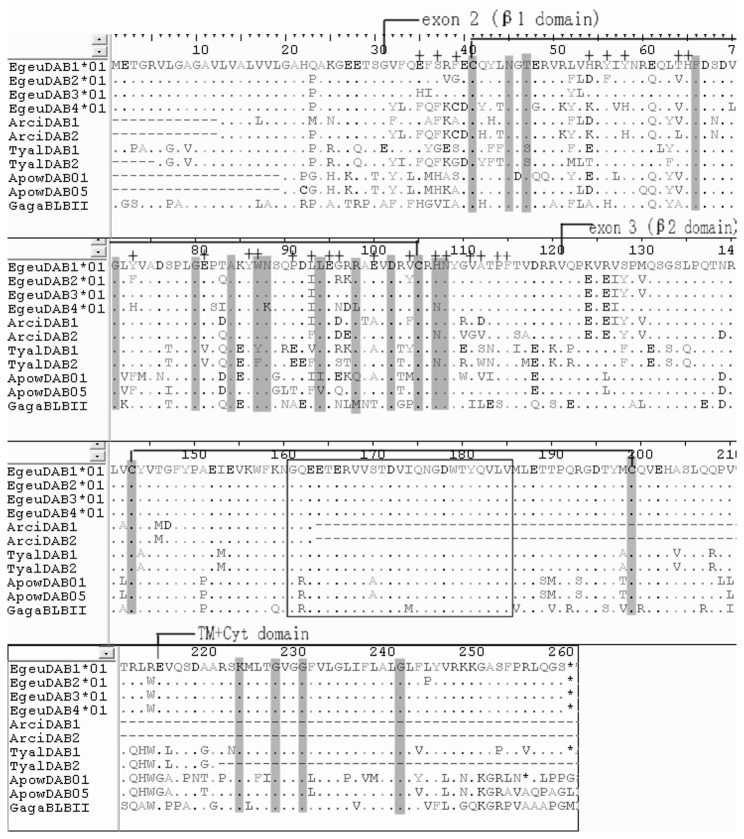
Amino acid alignment of transcribed Chinese egret MHC class II β chain sequences compared with those of other bird species. *Dots* indicate identity with the Egeu-DAB1*01 sequence, *dashes* indicate gaps. Conserved residues characteristic of classical class II β chain molecules [41] are *shaded*. Amino acids that contact the peptide-binding region in the human DRβ molecule [42] are indicated with a *plus sign* (on alignment top). Cysteine bridges in the β1 and β2 domains are indicated by *brackets*. Residues in the β2 domain that are implicated in CD4 binding in humans are boxed. Species and accession numbers for other bird sequences are as follows: ArciDAB1 Grey Heron, HM991016; ArciDAB2 Grey Heron, HM991017; TyalDAB1 Barn Owl, EU442606; TyalDAB2 Barn Owl, EU442607; ApowDAB01 kiwi, HQ639683, ApowDAB05 kiwi, HQ639685; GagaBLBII chicken, NM_001044679.

When expression analyses were carried out by RT-PCR in six individuals, a total of nine alleles were obtained after sequencing of the cloned RT-PCR products. Four of these alleles were derived from Egeu-DAB1, two from Egeu-DAB2, one from Egeu-DAB3 and two from Egeu-DAB4 (GenBank Accession KC282845-KC282853). The cDNA sequences contained features expected of functional class II molecules, and BLAST searches produced significant hits with known MHC DAB protein sequences. CD-Search identified a β1 domain in exon 2 and an IGc1 domain (β2 domain) in exon 3. SMART confirmed these domains, and identified a signal peptide and a trans-membrane domain. The manual survey confirmed the conserved intra-domain cysteine salt bridges within β1 and β2 domains (Figure 2). Additionally, the successful amplification from liver and blood shows that all four genes were expressed in these tissues.

### Locus Identification and Polymorphism

**Figure 3:**
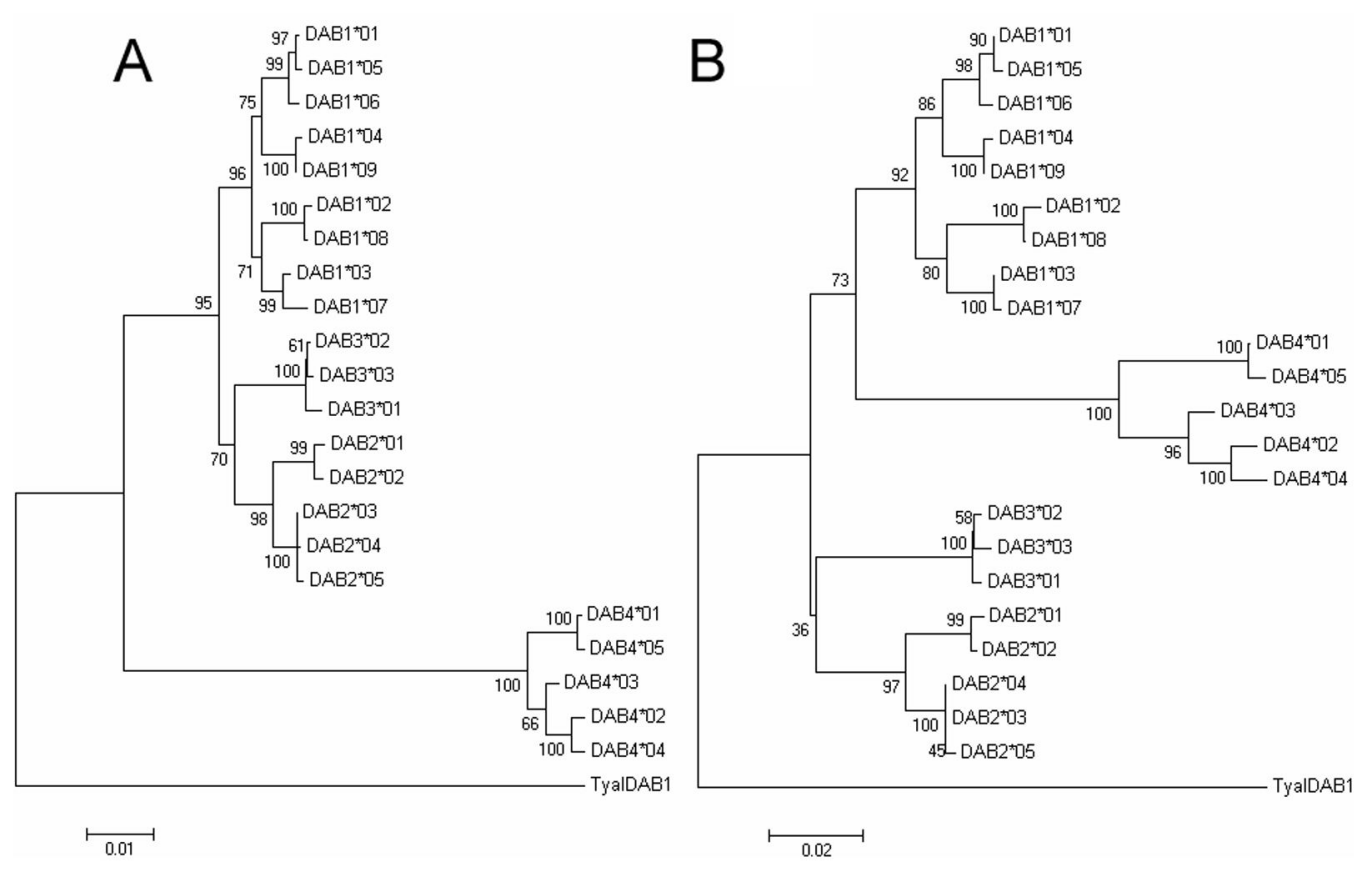
Neighbor joining tree of the 22 alleles obtained from 32 nestlings of the Chinese egret. The two trees are constructed with sequences from 5’ UTR to exon 3 (A) and only exons (B), respectively by using the MEGA 4.0 software program. A Barn Owl MHC class II sequence, TyalDAB1 (accession numbers EU442606), served as the outgroup.

Using the primers 25utrF1 and 23exr, 32 of 48 nestlings were amplified, and 22 sequences were obtained from independently replicated individuals. According to the sequence differences in intron 1, 9 of the 22 sequences were derived from Egeu-DAB1, 5 from Egeu-DAB2, 3 from Egeu-DAB3 and the remainder from Egeu-DAB4 (GenBank Accession KC282854-KC282875). This grouping was also confirmed by the neighbor joining analysis (Figure 3). Based on the clustering results, four forward locus-specific primers were designed on intron 1 for each locus, respectively. The SSCP results showed one or two alleles per individual on each locus, certifying the locus separation.

### Polymorphism in the MHC DAB Gene Complex

Complete four loci genotypes were obtained for 48 nestlings using a combination of SSCP and direct sequencing for each allele. Nineteen alleles in total were obtained from four loci, including 9 from Egeu-DAB1, 6 from Egeu-DAB2, 1 from Egeu-DAB3 and 3 from Egeu-DAB4 (GenBank Accession KF314166-KF314184). Among the four genes, Egeu-DAB1 had the greatest number of alleles and showed the most variability in nucleotide and amino acid sequences, whereas Egeu-DAB3 was monomorphic on exon 2 (Table 1 and Table S1).

**Table 1:** Summary of sequence variation of MHC DAB in the Chinese egret.

	**S_nt_**	**S_AA_**	**π**	**d_N_**	**d_S_**	**d_N_/d_S_**	**p**	**z**
PBR sites								
Egeu-DAB1	27	14	0.198±0.036	0.237±0.063	0.081±0.042	2.93	0.019	2.033
Egeu-DAB2	17	8	0.148±0.033	0.153±0.043	0.142±0.058	1.08	0.693	0.279
Egeu-DAB3	-	-	-	-	-	-	-	-
Egeu-DAB4	13	7	0.129±0.037	0.122±0.052	0.159±0.070	0.77	0.477	-0.634
Non-PBR sites								
Egeu-DAB1	32	18	0.067±0.012	0.068±0.018	0.057±0.022	1.19	0.650	0.455
Egeu-DAB2	16	12	0.042±0.011	0.043±0.012	0.042±0.022	1.38	0.981	0.024
Egeu-DAB3	-	-	-	-	-	-	-	-
Egeu-DAB4	18	10	0.065±0.015	0.053±0.018	0.076±0.037	0.70	0.598	-0.529

Total size 270 bp (90 residues) for all sites, 72 bp (24 residues) for PBR sites and 198 bp (66 residues) for non-PBR sites; S number of variable nucleotide (nt) or amino acid (AA) sites; π nucleotide diversity ± SE; d average rate of nonsynonymous (d_N_) or synonymous (d_S_) substitutions per site ± SE; z the Z test of positive selection.

### Frequency of Allele and Hardy-Weinberg Equilibrium

Our allele frequency results (Table 2) showed that alleles of each locus had an uneven distribution. On each locus there were one or two alleles had higher frequency than others, such as Egeu-DAB1*05, Egeu-DAB2*02, Egeu-DAB2*05 and Egeu-DAB4*01. The departure from Hardy-Weinberg equilibrium within each locus was statistically significant (P<0.001).

**Table 2:** Allele frequency of MHC DAB in the Chinese egret.

**Allele\ Locus**	**Egeu-DAB1**	**Egeu-DAB2**	**Egeu-DAB3**	**Egeu-DAB4**
01	0.0729	0.0833	1.0000	0.6250
02	0.0208	0.2917		0.2083
03	0.0208	0.0833		0.1667
04	0.1042	0.0833		
05	0.4167	0.2708		
06	0.1354	0.1875		
07	0.1042			
08	0.0417			
09	0.0833			

### Tests of Selection

When the ratio of non-synonymous to synonymous substitution (d_N_/d_S_) was analyzed in predicted peptide-binding regions sites (PBR) and remain sites (non-PBR) for each gene separately [42], significant signs of positive selection was only found in the PBR of Egeu-DAB1 alleles, with a d_N_/d_S_ ratio of 2.93 (Z test, P<0.05) (Table 1). Furthermore, it was tested if positive selection was acting on the exon 2 in the Chinese egret. The LRT statistic comparing M7 and M8 models indicated that M8 fitted the data significantly better than M7 (P<0.001). The estimates from M8 suggested that 17 amino acid sites of the exon 2 were under strong positive selection. As shown in Table 3, Egeu-DAB1 had the greatest number of residues with posterior probabilities of positive selection (P≥0.95). The peptide binding regions (PBR) were slight different from humans [42], but highly consistent with some birds, such as chicken, thin-billed prion and raptor [11,16].

**Table 3:** Log-likelihood values and parameter estimates of MHC DAB exon 2 alleles in the Chinese egret.

**Locus**	**Model**	**ln L**	**Parameter estimates**	**Positively selected sites**	**2△L(LRT)**
DAB01	M7	-829.2402	p= 0.00822, q= 0.01089	Not allowed	
	M8	-802.7880	p0=0.84765, p=0.00500, q=0.00753, (p1=0.15244), ω= 10.80638	**5** **, 7*, 8**, **9** **, 33**, **34** **, 53**, **66** **, 67**, 70*, 86**	52.9044(P<0.001)
DAB02	M7	-561.4464	p= 0.00745, q= 0.00500	Not allowed	
	M8	-554.4012	p0=0.88891, p=0.03131, q=0.02570, (p1=0.11109), ω= 15.72271	5**, 34**, 57**, 63*, 82**	14.0904(P<0.001)
DAB04	M7	-489.1666	p= 0.00500, q= 0.01175	Not allowed	
	M8	-481.6974	p0=0.79860, p=0.00500, q= 20.92618, (p1=0.20140), ω= 12.41712	27*, 42*, 43*, 53**, 66*, 82**	14.9384(P<0.001)

Sites inferred to be under positive selection at the 95% (*) and 99% (**) confidence interval level are indicated. lnL, log-likelihood value; ω, selection parameter; p_n_, proportion of sites that falls into ω_n_ site class. The sites consistent with human PBR sites are indicated in *bold*.

## Discussion

Using a PCR-based approach, four MHC DAB genes in the Chinese egret were isolated and characterized. Compared with the results of Li’s study [33], two loci, Egeu-DAB2 and Egeu-DAB3, were newly discovered in this egret. These four MHC DAB genes were all functional, based on the following findings: (1) they were expressed in several tissues; (2) some sites on PBR were under strong positive selection; (3) no frame shift mutations and stop codons were found in any coding region. These findings are thus in accord with the postulate that MHC organization is relatively simple, and contains rare pseudogenes in the nonpasserine birds [11,22,47,48].

The dominantly expressed ‘major’ chicken MHC II β-chain（B-LB) gene showing high sequence variation and high expression levels across tissue types, was previously described [22,49]. Among the four MHC DAB genes in the Chinese egret, genetic diversity in Egeu-DAB1 locus was higher than other three loci, and the number of alleles obtained from this locus by RT-PCR was more than that from the other three loci. Additionally, the d_N_/d_S_ values (all site or PBR site) suggested that Egeu-DAB1 locus was under positive selection. Therefore, these results suggest that Egeu-DAB1 may represent a major class II gene in the Chinese egret. In contrast to the greater sequence variation of Egeu-DAB1, Egeu-DAB3 showed extremely limited variation and was monomorphic in the PBR-site, suggesting this locus may represent a minor DAB gene [49]. Moreover, CODEML analyses revealed 17 residues with posterior probabilities of positive selection (P≥0.95) in MHC DAB exon 2, indicating that positive selection played a part in the adaptive evolution of this egret.

Many studies on characterization of the MHC gene complex have, as a major goal, identification of single MHC gene for population genetic studies. However, it is difficult to identify individual genes because the molecular mechanisms that underlie MHC evolution, including selection and concerted evolution, can obscure the genealogical relationships among alleles [50]. Segregation analyses using individuals with well-defined pedigrees may aid in clarifying the number of loci and assigning sequences to loci. However, such pedigree information is not available for wild birds such as the Chinese egret. Therefore, using noncoding and conserved regions as an indicator of which sequences representing individual loci, is an acceptable option, as it is less variable and not subject to balancing selection. Efforts to sequence noncoding regions of MHC loci can be rewarded by the ability to characterize individual loci, and can enhance the utility of these genes as molecular markers in evolutionary ecology research. This experimental strategy also contributes greatly to the study of MHC gene family evolution across the breadth of avian diversity. In this study, intron 1 was used to show that this intron was more divergent than the other regions of the MHC DAB genes, suggesting it may be the best candidate for locus specific primers in single locus typing of MHC class II in the Chinese egret. The low number of loci in this egret will allow us to completely genotype each locus of the MHC II B in individual egrets. Moreover, the primers designed for this study are targeting highly conserved regions across class II genes, and therefore, similar fragments in other Ardeid species are likely to be cross-amplified successfully.

## Supporting Information

Table S1 **Genotyping data collected from 4 MHC DAB loci in the Chinese egret.** The null alleles are indicated by dots.
(DOC)
