# Supplementary material for: Characterization, Polymorphism and Selection of Major Histocompatibility Complex (MHC) DAB Genes in Vulnerable Chinese Egret (Egretta eulophotes)
Source: PLoS One. 2013 Sep 3;8(9):e74185. doi: 10.1371/journal.pone.0074185 (PMC3760844; doi:10.1371/journal.pone.0074185)
Supplement: Table S1 — Genotyping data collected from 4 MHC DAB loci in the Chinese egret. The null alleles are indicated by dots. (DOC) [file pone.0074185.s001.doc]

**Table S1. Genotyping data collected from 4 MHC DAB loci in the Chinese egret.**

| **Individual\Locus** | **Egeu-DAB1** | **Egeu-DAB2** | **Egeu-DAB3** | **Egeu-DAB4** |
| --- | --- | --- | --- | --- |
| 1 | 0505 | 0206 | 0101 | .. |
| 2 | 0506 | 0505 | 0101 | 0101 |
| 3 | 0407 | 0206 | 0101 | 0102 |
| 4 | 0507 | 0206 | 0101 | 0102 |
| 5 | 0506 | 0104 | 0101 | 0101 |
| 6 | 0505 | 0205 | 0101 | 0101 |
| 7 | 0505 | 0205 | 0101 | 0101 |
| 8 | 0505 | 0303 | 0101 | .. |
| 9 | 0505 | 0303 | 0101 | .. |
| 10 | 0407 | 0202 | 0101 | .. |
| 11 | 0101 | 0104 | 0101 | .. |
| 12 | 0101 | 0202 | 0101 | .. |
| 13 | 0102 | 0104 | 0101 | .. |
| 14 | 0808 | 0104 | 0101 | .. |
| 15 | 0304 | 0104 | 0101 | .. |
| 16 | 0407 | 0206 | 0101 | .. |
| 17 | 0506 | 0206 | 0101 | 0303 |
| 18 | 0505 | 0206 | 0101 | .. |
| 19 | 0506 | 0206 | 0101 | .. |
| 20 | 0506 | 0206 | 0101 | 0203 |
| 21 | 0506 | 0206 | 0101 | 0203 |
| 22 | 0407 | 0206 | 0101 | 0101 |
| 23 | 0407 | 0206 | 0101 | .. |
| 24 | 0407 | 0206 | 0101 | .. |
| 25 | 0707 | 0206 | 0101 | .. |
| 26 | 0808 | 0506 | 0101 | .. |
| 27 | 0505 | 0505 | 0101 | .. |
| 28 | 0101 | 0505 | 0101 | .. |
| 29 | 0506 | 0206 | 0101 | 0101 |
| 30 | 0506 | 0505 | 0101 | .. |
| 31 | 0505 | 0505 | 0101 | .. |
| 32 | 0505 | 0505 | 0101 | .. |
| 33 | 0909 | 0202 | 0101 | .. |
| 34 | 0505 | 0505 | 0101 | .. |
| 35 | 0407 | 0303 | 0101 | .. |
| 36 | 0505 | 0303 | 0101 | .. |
| 37 | 0505 | 0205 | 0101 | .. |
| 38 | 0204 | 0104 | 0101 | .. |
| 39 | 0505 | 0205 | 0101 | 0102 |
| 40 | 0505 | 0205 | 0101 | .. |
| 41 | 0506 | 0206 | 0101 | .. |
| 42 | 0506 | 0104 | 0101 | .. |
| 43 | 0506 | 0104 | 0101 | .. |
| 44 | 0304 | 0206 | 0101 | .. |
| 45 | 0909 | 0505 | 0101 | .. |
| 46 | 0909 | 0505 | 0101 | .. |
| 47 | 0909 | 0505 | 0101 | .. |
| 48 | 0606 | 0206 | 0101 | .. |

The null alleles are indicated by *dots*.
